# Selective targeting of myeloid-derived suppressor cells in cancer patients through AFP-binding receptors

**DOI:** 10.4155/fsoa-2018-0029

**Published:** 2018-06-28

**Authors:** Vladimir N Pak

**Affiliations:** 1 Freelance Researcher, Toronto, M2R 3T1, Canada

**Keywords:** AFP-toxin drug, alpha-fetoprotein (AFP) receptor, immunotherapy, myeloid-derived suppressor cells

In theory, a mother during pregnancy (especially a surrogate mother) is ‘infected’ by an alien embryo, yet her immune system does not reject it. The fetus and placenta express antigens, which are foreign to the mother's system. These come into direct contact with cells of the maternal immune system, but fail to provoke the tissue rejection response that is observed with, for example, organ transplants. Embryo implantation turns off a key pathway required for the immune system to attack foreigners. To suppress the immune response, the embryo generates AFP and other glycoproteins that inhibit rejection of the fetus. Immunity suppression is also common in autoimmune and infectious diseases, cancer, transplantation, etc.

Among the major participants in the mechanism of immune suppression are myeloid-derived suppressor cells (MDSCs) [1]. It was a considerable discovery that myeloid(M)-MDSCs and granulocyte-MDSCs express AFP-binding receptor (AFPR) [2]. Previously, AFPR was considered only as a cancer cell marker. Similar to embryo cells, MDSCs are supplied with nutrients delivered by embryo-generated transport protein–AFP. This way MDSCs are activated to suppress the mother's immune reaction to the embryo during pregnancy [3]. The AFP–AFPR nutrient delivery system stimulates MDSCs to suppress both innate and adaptive immunity.

The same mechanism enhances the tumor's ability to survive inside its host. Tumor cells generate an immunosuppressive tumor microenvironment, which inhibits antitumor immune responses and supports disease progression. It is now well established that MDSCs play an important role in regulation of tumor progression and metastasis as well as in limiting the effects of cancer immunotherapy. Background information surrounding MDSCs, including their physiological functions and their role in cancer proliferation, known regulators of MDSCs, and notable development in cancer research related to MDSC targeting, are well-covered in the literature, for example, in [1].

Heterogeneous subpopulations of immature myeloid cells inhibit antitumor activity of natural killer (NK) and T cells, recruit immunosuppressive T-regulatory cells, and promote angiogenesis. Innate immunity NK cells act as the first line of defense in the body. They can destroy infected, low-differentiated and cancer stem cells. Unlike T cells, NK cells recognize and attack foreign material without having been exposed to it previously. Therefore, the uncontrolled spread of cancer cells, metastasis, is a result of a fault in innate immunity [4]. MDSC activation correlates with tumor progression, recurrence, and negative clinical outcome [5]. So, the elimination of MDSC-induced immune suppression is an urgent oncology problem.

On the other hand, MDSC depletion can reverse immune suppression. Human MDSCs can be differentiated from other immune cells and possibly depleted by antibodies targeted to their specific antigens: CD11b, CD14, CD15 and CD33, among others [5].

Successful specific MDSC depletion in cancer patients was recently shown with agonistic TRAIL receptor antibody [6].

Alternatively, MDSC-specific antibodies can be generated by vaccination. Vaccination of cancer patients with AFP, AFPR and AFP–AFPR complexes can raise an immune response that depletes AFPR-positive MDSCs (as well as cancer cells). This enables the innate and adaptive immunity to destroy cancer cells and metastases [7]. This ‘Achilles-heel’ of cancer [8] was discovered and used by Govallo who achieved a 77.1% 5-year survival rate, and a 65.4% 10-year, or more, survival rate in 35 terminal patients after vaccination with the placental proteins [9], the majority of which comprised oncofetal AFP and AFPR.

An increase of antibodies to AFPR was detected in patients with malignant tumors, which indicates the induction of a primary immune response to AFPR in such patients. Separate clones of antibodies to AFPR are capable of activating the immune system in respect to AFPR-positive cells, inducing antibody-dependent cellular cytotoxic activity. In the case of single immunization of mice with an AFPR preparation, isolated from tumor tissue of human lung cancer, inhibition of the growth of a tumor, grafted 4 days after the immunization, was observed [10].

Anticancer vaccinations with AFP-AFPR in humans could be possible once recombinant AFP and AFPR become available on the pharmaceutical market.

Antibodies to AFPR (manufactured as anti-idiotypic to antibodies to AFP) have been patented under the name ‘Normogen’ as a preparation for cancer treatments. The authors reported that ‘Normogen’ antibody preparation monotherapy improved the patient's quality of life [11].

MDSCs can be depleted through AFP-binding receptors by an AFP-toxin drug. An AFP covalent conjugate with daunorubicin has been shown to be able to specifically deplete G- and M-MDSCs [2]. NK and T cells were released to attack cancer cells, as these are subordinate to MDSCs [12].

MDSCs can be depleted in a natural way through AFP-binding receptors by AFP–toxin noncovalent complex. For example, AFP-amphotericin B complex was targeted to AFPR-positive cells and demonstrated response to treatment in six out of eight cancer patients [13]. Acute-phase immune response has sometimes been seen during the complex infusions that can indicate MDSC depletion. The high efficacy of the extremely low doses of AFP suggests that the main driver of cancer cells death is rather MDSC depletion and subsequent immune system activation than the direct action of the complex on AFPR-positive cancer cells. Moreover, a 50% response rate was demonstrated in 12 metastatic colorectal cancer patients with AFP–atractyloside complex administrated orally in low doses [14]. Taking into account the inability of the AFP–toxin complex to be absorbed from the GI tract into the blood in an active form (unpublished data) and to reach liver metastases, we assume that it can deplete MDSC-like cells in Payer's patches [12]. In this case, unleashed subordinate NK and T cells could attack nearby and rare metastases. An oral route of the AFP-drug noncovalent complexes administration and their possible interaction in Payer's patches with immune cells needs additional research.

Prospective AFP noncovalent complexes with 1′-S-1′-acetoxychavicol acetate [15] and taxane [16] are awaiting clinical trials. In my opinion, the trials should be successful because AFP and toxin (e.g., the registered drug paclitaxel) doses needed for immunotherapy are much lower than those used for chemotherapy (for which safety ranges were proven in clinical trials before registration). It should, however, be taken into account that only patients with uncompromised (or restored after chemotherapy) immune systems could be provided with AFP-toxin complexes for anticancer treatments. The close monitoring of myeloid and other immune cell balance during treatments would also be necessary.

Chemokine CCR5 is also an AFP-binding receptor [17] and hence, an AFP–toxin noncovalent complex could possibly hit M-MDSCs through this receptor (as well as CCR5-positive T-regulatory cells). Targeting the CCR5 could prevent MDSC accumulation and suppress tumor growth in pancreatic, colorectal, prostate, and breast cancer [5].

An MDSC depletion strategy activates innate and adaptive immunity and represents a new way to direct immune response against cancer [5]. Use of NK cells to attack metastases could be an obligatory addition to modern T cells. AFP–toxin complexes could be used for metastases prophylactics and diagnostics at the same time [12] and understanding efficacy of complexes against metastases is the next step in this research field.

AFP-toxin complexes that selectively target MDSCs through AFP-binding receptors could become a necessary tool for cancer/metastases treatments and could be used as monotherapy or combined with other immunotherapies and chemotherapies. As a matter of fact, the AFP–toxin complex itself works as the combination of MDSC-targeted immunotherapy and cancer cells-targeted chemotherapy [7] and is remarkably promising. Unlike T-cell immunotherapies, MDSC-targeted immunotherapy is not personalized and consequently could be more affordable for cancer patients.

MDSCs are at the top of the regulatory immune cell hierarchy, and their precise tuning is a hot topic of today's research into cancer, autoimmune and infectious diseases, transplantation, pregnancy and other immunology-related fields. Overall, MDSC AFP-binding receptors could be successful in the treatment of autoimmune diseases as well as cancer treatment [18].
